# Oral Complications in Cancer Patients–Medication-Related Osteonecrosis of the Jaw (MRONJ)

**DOI:** 10.3389/froh.2022.866871

**Published:** 2022-04-26

**Authors:** Cesar Augusto Migliorati

**Affiliations:** Department of Oral and Maxillofacial Diagnostic Sciences, Division of Oral Medicine, University of Florida College of Dentistry, Gainesville, FL, United States

**Keywords:** ONJ, MRONJ, osteonecrosis of the jaw, osteonecrosis, oral complications, cancer therapy

## Abstract

Medication-Related Osteonecrosis of the Jaw (MRONJ) was first reported in 2003. Despite the progress in the understanding of this oral complication in cancer patients for the past 18 years, there is still discussion about the best way to define MRONJ, prevent the complication, how to diagnose, and the options of treatment available. The initial reports associated MRONJ to bisphosphonates and denosumab, medications that work as bone-modifying agents. Later, other agents such as the antiangiogenics, have also been reported to cause the oral complication, either alone or in combination with antiresorptives. Initially, these medications were prescribed to patients with osteoporosis and cancers patients with bone metastasis. Today, because of the effect of the medications in the bone remodeling system, patients with several other diseases such as giant cell tumors, rheumatoid arthritis, Paget's disease of bone, fibrous dysplasia, osteogenesis imperfecta, are managed with these medications, significantly increasing the population of individuals at risk for developing MRONJ. This mini review focused on the cancer patient. It updates the dental clinician on the recent scientific literature about MRONJ and provides information on how to diagnose and manage patients being treated with these medications, suggests protocols to prevent the development of MRONJ, and present ways to manage those patients who develop the oral complication.

## Introduction

The history of MRONJ started about 18 years ago when it was first reported [1–3]. After many suggestions to name the complication, including ONJ, BON, BIONJ, ostechemonecrosis, BONJ, and many others, the final universal agreement came with a proposal from the clinical guidelines article from the American Academy of Oral and Maxillofacial Surgeons (AAOMS) in 2014 that named it “medication-related osteonecrosis of the jaws, or MRONJ” [4] due to its association with medications. This is the terminology we will use throughout this mini review. Despite the progress in the understanding of this oral complication in patients with cancer, there is still discussion about the best way to define, diagnose, and stage MRONJ, the mechanisms that lead to the development of the oral complication, management alternatives with medical or surgical interventions, and best prevention measures. We will discuss current knowledge about patient management, with the goal of assisting the dental provider when treating patients, taking drugs reported to be associated with MRONJ and those patients who develop the complication.

## Methods

We conducted a brief PubMed review of the recent literature, addressing MRONJ in patients with cancer. Using the key words bisphosphonates and osteonecrosis, the initial search revealed 3,635 publications from 2003 through 2021. From this search, relevant articles written in English were reviewed, and pertinent information was collected. When available, clinical trials were used as the main source of information. Otherwise, important research and personal expert experience developed during the past 18 years will be used. A recent joint clinical practice guidelines manuscript published by the Multinational Association of Supportive Care in Cancer, the International Society of Oral Oncology and the American Society of Clinical Oncology (MASCC/ISOO/ASCO) has revealed a lack of robust clinical trials, making difficult to produce guidelines based solely on scientific evidence [5].

### Mini-Review Results

#### Definition, Diagnosis, and Clinical Staging

MRONJ is an oral manifestation characterized by exposed necrotic jawbone of patients who are using one of the medications that have been associated with the complication. To diagnose this condition, the clinician should confirm the presence of the three following criteria [4, 5]:

- Current or previous treatment with bone-modifying agents, such as a bisphosphonate, denosumab, or an antiangiogenic.
- The presence of an exposed necrotic bone or a bone that can be probed through an intraoral or extraoral fistula in the maxillofacial region and that has persisted for longer than 8 weeks.
- No history of radiation therapy to the jaws or obvious metastatic disease to the jaws.

**Figure 1:**
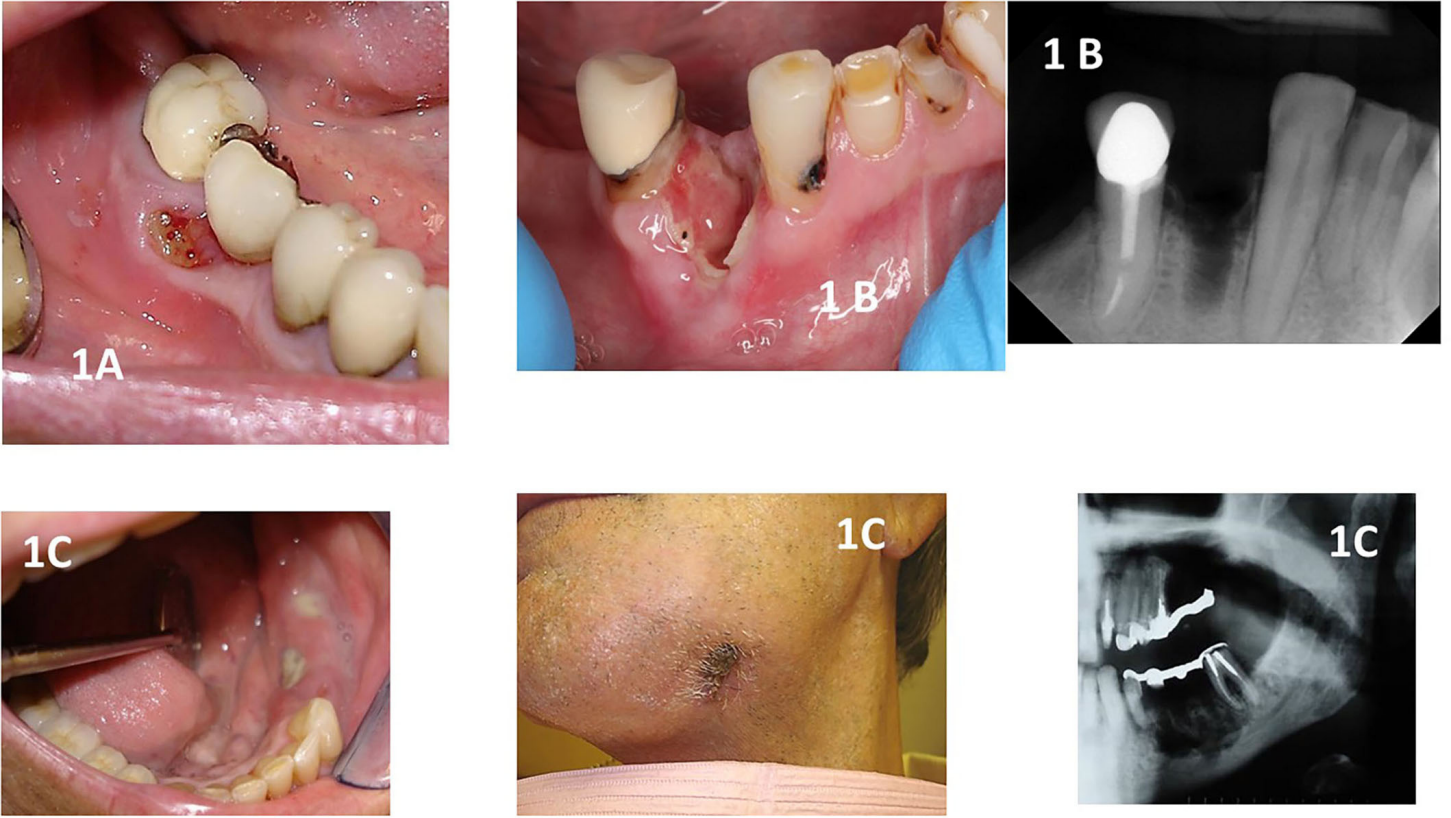
Shows clinical and radiographical images of different stages of MRONJ. **(A)**: stage 1 showing a patient with breast cancer taking zoledronic acid with a small asymptomatic area of exposed necrotic bone that was affecting the use of a removable prosthodontic appliance. **(B)**: shows a patient taking denosumab who had a recent dental extraction. The site was not healing, and exposed alveolar bone could be seen. The patient was in pain and did not respond to antibiotics. The radiographical image shows the non-healing alveolus. **(C)**: shows a patient with multiple myeloma with Stage 3 MRONJ. The patient was in pain and presented to the clinic with swelling of the alveolar mucosa and several areas of infected and exposed bone with pus drainage. The patient complained of paresthesia in the area and had a strong mal odor. One can observe an extraoral fistula on the left submandibular area. The radiograph shows the extensive area of bone destruction, placing the patient at risk for a pathological fracture. Note that the fixed prosthodontic appliance was removed, revealing the exposed bone seen in this figure.

In addition to a precise definition and diagnosis, one should stage the complication before management is proposed (Figure 1). Staging of MRONJ is of importance in the decision-making process on how to manage each of the patients. The staging classification proposed in 2014 by the AAOMS guidelines article established the following staging criteria (updated based on new evidence):

#### At Risk

No apparent necrotic bone in patients who have been or who are being treated with oral or intravenous antiresorptives and or antiangiogenics.

#### Stage Zero

No clinical evidence of a necrotic bone but nonspecific clinical findings, radiographic changes, and symptoms. This staging terminology is controversial and may lead to under or overdiagnosis MRONJ. Clinicians should be aware of this possibility when diagnosing patients at risk for MRONJ [6–8].

#### Stage 1

Exposed and necrotic bones or fistulas that probe to be bones in patients who are asymptomatic and have no evidence of infection.

#### Stage 2

Exposed and necrotic bones or fistulas that probe to be bones associated with infection as evidenced by pain and erythema in the region of exposed bones with or without purulent drainage.

#### Stage 3

Exposed and necrotic bones or a fistula that probes to be a bone in patients with pain, infection, and ≥ 1 of the following: exposed and necrotic bones extending beyond the region of alveolar bone (i.e., inferior border and ramus in mandible, maxillary sinus, and zygoma in maxilla), resulting in pathologic fracture, extraoral fistula, oral antral or oral nasal communication, or osteolysis extending to the inferior border of the mandible or sinus floor.

An important aspect of the staging system is presented by the Italian consortium on MRONJ, stating that imaging examination is necessary to precisely diagnose and stage the oral complication [9]. Areas of osteosclerosis and bone changes can assist the clinician in determining the real extension of the complication and help in the planning of management [10, 11].

### Prevalence of MRONJ

How prevalent is MRONJ among patients being treated with one of the drugs associated with the oral complication? The prevalence is small. For the dental provider, it is important to know that patients with cancer have a higher risk of developing the complication than patients who use the medications for osteoporosis. A prospective controlled study compared zoledronic acid (ZA) and denosumab use in 5,723 patients with cancer. The overall risk of MRONJ was 1.6%. In patients being treated with zoledronic acid, 1.3% developed MRONJ, whereas 1.8% of patients treated with denosumab developed MRONJ [12]. A review from 2008 to 2015 suggested that the frequency of MRONJ is about 1% (range, 2–6.7%) [13]. The recommendation for the clinician is to consider that any patients exposed to the medications are at risk to develop the complication.

### What Are the Drugs That Have Been Reported to Be Associated With MRONJ?

The first group of drugs associated with MRONJ were the bisphosphonates Pamidronate (Aredia^®^) and zoledronic acid (Zometa^®^). These drugs inhibit osteoclasts and are used to treat patients with cancer with malignancies that metastasized to bones, such as multiple myeloma, breast, prostate, and lung [1, 2, 14]. Following, with the development of denosumab (XGeva^®^), a humanized monoclonal antibody with similar action over osteoclasts, new cases of MRONJ were reported. Currently, several other drug groups have been associated with MRONJ, including the antiangiogenics, targeted therapy, and biologic immunomodulators [15–17]. The use of these drugs places individuals at risk for the development of MRONJ. The clinician must certify if a patient is being treated with one of the drugs when reviewing medical history so a prevention protocol can be used during patient care. It has been proposed that patients with cancer being treated with a combination of bisphosphonates and antiangiogenics may be at increased risk for MRONJ [18].

### Common Signs and Symptoms Associated With MRONJ

The most common signs and symptoms associated with MRONJ observed in patients who have developed the complication include pain, infection with purulent secretion, general jaw discomfort, paresthesia, mal odor, a non-healing extraction site, or a sore associated with an ill-fitting denture [4, 5]. Of major importance is the presence of exposed necrotic bone or bone that could be probed through a fistula, according to the currently accepted definition of MRONJ [4].

It has been postulated that clinicians who manage patients considered at risk for MRONJ would benefit from having a diagnostic test that would indicate increased risk for MRONJ prior to doing invasive dental care. A study has hypothesized that bone remodeling markers may be indicators of the risk of development of MRONJ [19]. However, there is controversy in the literature whether or not such bone remodeling markers may, indeed, indicate risk [20]. A more recent study using different markers of bone changes in patients taking an antiresorptive medications [21] has evaluated 12 different biomarkers in patients with and without MRONJ. They suggested that tartrate-resistant acid phosphatase isoform 5b (TRACP 5b) levels were significantly lower, and the mean Dickkopf-related protein 1 (DKK1) levels were significantly higher than the corresponding values for the control group (without MRONJ). This indicates the need to carefully follow patients with these abnormal biomarkers before and after dental extractions. However, one must always consider the availability of such tests and the cost of running them.

### What Is the Mechanism That Leads to MRONJ?

A large body of research has been published in order to establish the mechanism that leads to the formation of MRONJ. The current evidence is that MRONJ is a multifactorial complication resulting from the effect of antiresorptive drugs-inhibiting osteoclasts and altering the bone remodeling system [22], the presence of dental infection both in the periodontium and the periapical areas [23, 24], chronic inflammation and acidic environment [25], dental trauma from dental extractions or invasive surgery [26], diabetes and other chronic disease [27], the use of corticosteroids, and altered local immunity [28]. This continues to be researched with the goal of determining the precise mechanism that results in MRONJ. However, there is no doubt that the antiresorptives and the antiangiogenics play a very important role. There is also evidence that the combined use of bisphosphonates and antiangiogenics in certain types of cancer increases the risk for the complication [29].

### Suggestions of Management Protocols

#### Patients at Risk for MRONJ and Prevention Protocols

Patients who will be prescribed medications associated with MRONJ should have their oral health stabilized as soon as possible, preferably prior to the start of the drug therapy. A complete evaluation of teeth, periodontium and radiographic examination should be done. The dentist should perform dental extractions of hopeless teeth, scaling and root planning, dental restorations, and implement good oral hygiene. There is evidence that this may prevent or decrease the risk of MRONJ development [5, 6, 30, 31]. A periodic follow-up could be planned, depending on the oral health of each of the patients. If the drug therapy has started, dental procedures can be planned together with the patient's physician. Patients with complex medical conditions may not be exposed to invasive surgical procedures, and a more conservative dental care should be done. One must always consider the best alternative for the overall health of the patient. Although MRONJ is a severe complication, the risk is relatively small. Patients with active dental and periodontal infections may benefit from local treatment to control the infection associated with antibiotic therapy and topical antiseptic rinses [5, 32].

#### Patients With MRONJ

Dental professionals with expertise in managing MRONJ should be the ones to treat patients with this complication [5]. It is recommended that the decision on how to treat the patient should be done by a multiprofessional team. MRONJ must be staged, the overall medical health status evaluated, and the prognosis of the cancer should be considered. In general, experts propose two modalities of therapy: medical and surgical treatments. However, controversy exists in the literature about which is the best approach [17, 33].

Medical treatment is a more conservative approach. Minor local debridement of areas of exposed necrotic bone can be performed, sharp edges of bone can be eliminated, and active infection managed with antibiotics and topical antibacterial rinses. Some have proposed the use of a combination of pentoxifylline and tocopherol (vitamin E) such as it is done with patients with osteoradionecrosis [34]. However, clinical and radiographic results may take months or years to happen [35, 36]. Patients who cannot use pentoxifylline, cilostasol can be an alternative [37]. Conservative therapy can also be of value to treat patients ineligible to surgery [38].

Surgical treatment as the first choice for the treatment of MRONJ has been proposed regardless of staging [39]. The surgical approach must aim to the removal of all necrotic bones and closure by primary intention [40]. Several studies have shown improvement and resolution of MRONJ with surgical management [41, 42]. Surgical lasers have been used with reasonable success [43, 44].

## Discussion

MRONJ is a relatively new oral complication in patients with cancer being treated with medications used in cancer care. We provided current information that may be used by the clinician when managing patients at risk or with MRONJ. However, professional expertise in the diagnosis, staging, and management of patients with MRONJ is of importance for the success of the treatment [5].

It is suggested that a team of medical and dental providers must make all decisions about the best way to manage patients at risk for MRONJ. The idea of the patient discontinuing drug therapy is always present in the clinician's mind. However, the evidence available about a drug holiday prior to invasive dental procedures is not robust [45]. Discontinuation of antiresorptives may lead to more serious complications, such as skeletal-related fractures.

We revised information on the important aspects of taking complete dental and medical histories, detecting signs and symptoms that lead to the suspicion of MRONJ diagnosis and a patient's characteristics that help to determine risk of MRONJ development. We provided basic guidance for the clinician on current proposed prevention and management protocols.

### Research Gaps and Future Research Needs

There still exist research gaps that are being investigated by several authors. These gaps provide future research ideas in the field of MRONJ. For example, we still do not know the exact mechanism that leads to the formation of MRONJ. There are several suggestions, but the only thing that can be stated is that it is a multifactorial process [28]. However, what starts MRONJ and how the many mechanisms interact have not been established. There is a lack of information about the mechanisms involved in MRONJ with new, non-antiresorptive drugs that are being reported to be associated with the complication [16]. None of the proposed treatment protocols have been demonstrated to have complete success, but only partial resolution of MRONJ cases [17, 46]. There is a need to better understand the role that osteoimmunity plays [28] and whether or not a genetic predisposition exists among populations [47].

The final take-home message is that patients with cancer are prescribed drugs that help mitigate the burden of oncological disease. As any drug, some are associated with the development of MRONJ but have other beneficial effects. Individual patients have different kinds of cancer staging and prognosis, and only their oncologists can determine the risk of discontinuing a medication or of doing an invasive procedure in the oral cavity. It is common for patients with cancer to have additional comorbidities that may preclude invasive surgical procedures. The recommendation for the clinicians is to work in collaboration with the oncology team when making decisions to treat dental disease in this population of patients.

## Ethics Statement

Written informed consent was obtained from the individual(s) for the publication of any identifiable images or data included in this article.

## Author Contributions

The author confirms being the sole contributor of this work and has approved it for publication.

## Conflict of Interest

The author declares that the research was conducted in the absence of any commercial or financial relationships that could be construed as a potential conflict of interest.

## Publisher's Note

All claims expressed in this article are solely those of the authors and do not necessarily represent those of their affiliated organizations, or those of the publisher, the editors and the reviewers. Any product that may be evaluated in this article, or claim that may be made by its manufacturer, is not guaranteed or endorsed by the publisher.
